# Comparison of antibacterial effect of plantaricin 149 suspension and traditional root canal irrigation solutions in root canal infections in vitro

**DOI:** 10.1186/s12903-022-02683-4

**Published:** 2022-12-26

**Authors:** Ying Wang, Keda Chen, Xiaolong Lin

**Affiliations:** 1 Department of Endodontics, Stomatology Hospital, School of Stomatology, Zhejiang University School of Medicine, Zhejiang Provincial Clinical Research Center for Oral Diseases, Key Laboratory of Oral Biomedical Research of Zhejiang Province, Cancer Center of Zhejiang University,, Hangzhou, 310000 Zhejiang China; 2 Shulan International Medical College, Zhejiang Shuren University, Hangzhou, 310000 China

**Keywords:** Antibacterial effect, Antimicrobial peptide, Root canal irrigation solutions, Cytotoxicity

## Abstract

Dental pulp and periapical diseases are common conditions in stomatology, caused by various pathogenic microorganisms. Antimicrobial peptides, as new antibiotics, offer promising applications in the irrigation and disinfection medicaments for root canals.

One patient with chronic periapical periodontitis was selected to extract the clinical pathogenic bacteria. *Porphyromonas gingivalis* (Pg) (ATCC 33,277), *Streptococcus mutans* (Sm) (ATCC 25,175), and *Prevotella intermedius* (Pi) (ATCC 25,611) were used as test strains. The effects of plantaricin (Pln) 149 on the biofilm formation and growth in infected root canals were evaluated by RT-PCR, laser confocal scanning microscopy, and bacterial diversity analysis. In addition, the cytotoxicity of Pln 149 (100 µg/mL) to human dental pulp stem cells (hDPSCs) was assessed using an MTT assay. Pln 149 exhibited significant inhibitory effects on Pg Sm and Pi (P < 0.05), with significant differences in the biofilm images of the laser confocal scanning microscope (P < 0.05). There were no significant differences in hDPSCs viability or proliferation between the Pln 149 and control groups. Considering the excellent antimicrobial effects and low cytotoxicity, we suggest that Pln 149 might be a promising option for root canal irrigation solutions.

## Introduction

Dental pulp and periapical diseases are caused by bacterial infections [1]. Studies have shown that the colonization of obligate anaerobic and facultative anaerobic bacteria in root canals plays a vital role in the development of periapical periodontitis [2]. The microbiome of the infected root canals is a complex community comprised of bacterial species selected by specific environmental pressures and organized in biofilms [3]. At present, the primary treatment of chronic periapical periodontitis in the clinic is root canal treatment (RCT) [4]. However, the anatomical structure of the root canal system is complex, such as the apical constriction area and apical triangle [5]. Therefore, root canal preparation instruments cannot reach all parts of the root canal system. Root canal flushing and disinfection enhance the cleaning effect of endodontic instruments in the root canal system [6]. Root canal flushing fluids not only do play a role in flushing and lubricating the root canal, but also they have a certain bactericidal effect [7]. They play an important role in root canal cleaning and disinfection. Root canal irrigation is an indispensable step in during root canal preparation. At present, the commonly used root canal irrigation solutions in the clinic mainly include sodium hypochlorite, hydrogen peroxide, antibiotics, chlorhexidine, citric acid, and povidone-iodine [8]. Although many clinical studies [9] have shown that root canal preparation combined with sodium hypochlorite, hydrogen peroxide, and povidone-iodine root canal irrigation solutions can reduce bacterial counts in the root canal, antimicrobial peptides as new antibiotics have already shown strong antibacterial effects compared with traditional root canal irrigation solutions [10]. Plantaricin 149 (Pln149), is a cationic antimicrobial peptide produced by Lactobacillus plantarum NRIC 149 that has been identified as a bacteriocin and presents inhibitory spectrum on *Listeria* and *Staphylococcus strains*. Pln149 exert its antibacterial action by destory biomembrane model systems [11].

This study aimed to investigate the antibacterial effect of Plantaricin (Pln) 149 on root canal inflammation pathogens in vitro and test its cytotoxicity. For this purpose, we isolated the strains from the infectious root canal in one patient and tested bacterial changes and diversity using laser confocal scanning microscopy to verify the effects of Pln 149 on infectious root canals. In addition, its cytotoxicity on pulp stem cells was tested to verify the clinical value of Pln 149 in root canal therapy.

.

## Materials and methods

### Bacterial isolation and test of strains

The project was approved by the Ethics Committee of Zhejiang University School of Stomatology, Hangzhou. The following were used as inclusion criteria: Patients with chronic apical periodontitis with closed apical foramen and Age 18–35 years old. After obtaining written informed consent from the patient, one patient with chronic periapical periodontitis of mandibular premolars was selected at the Affiliated Hospital of Stomatology to extract the clinical pathogenic bacteria. Mouth rinse with 0.2% chlorhexidine (Lircon, China) for 3 min was used before rubber dam application. After sterilized with 75% alcoholic cotton ball, occlusal endodontic access cavities were prepared with a high-speed dental turbine to expose root canal orifices and allow a straight- line access of instruments. 1 ml of bacterial specimens were obtained by sterile syringe after root canal irrigation by saline solution, and then the 1ml bacterial specimens were added to 9 ml brain-heart infusion broth (BHI) (Difco, USA) and cultured under anaerobic conditions for three days. *Porphyromonas gingivalis* (Pg) (ATCC 33,277), *Streptococcus mutans* (Sm) [12] (ATCC 25,175), and *Prevotella intermedius* (Pi) (ATCC 25,611) were purchased from ATCC. Cultures of Pg, Sm, and Pi were grown overnight in BHI under anaerobic conditions (80% N_2_, 10% CO_2_, and 10% H_2_) at 37 °C in an anaerobic chamber.

### Peptide

Pln 149 (YSLQM GATAI KQVKK LFKKK GG) was synthesized by Shanghai Apeptide Co. Ltd (Shanghai, China). The peptide was purified by high-performance liquid chromatography, and its identity was verified by SDS-PAGE. The purity of Pln 149 (> 95%) and its mass were confirmed by electrospray ionization mass spectrometry [13].

### Minimum inhibitory (MIC) and minimum bactericidal concentration (MBC)

The MIC and MBC of Pln 149 with activity against Pg, Sm, and Pi strains were determined by applying different concentrations of the Pln 149 alongside the same bacterial loads in a nutrient broth. MIC and MBC were determined via the broth microdilution method [14].

### Infected root canal model

**Fig. 1:**
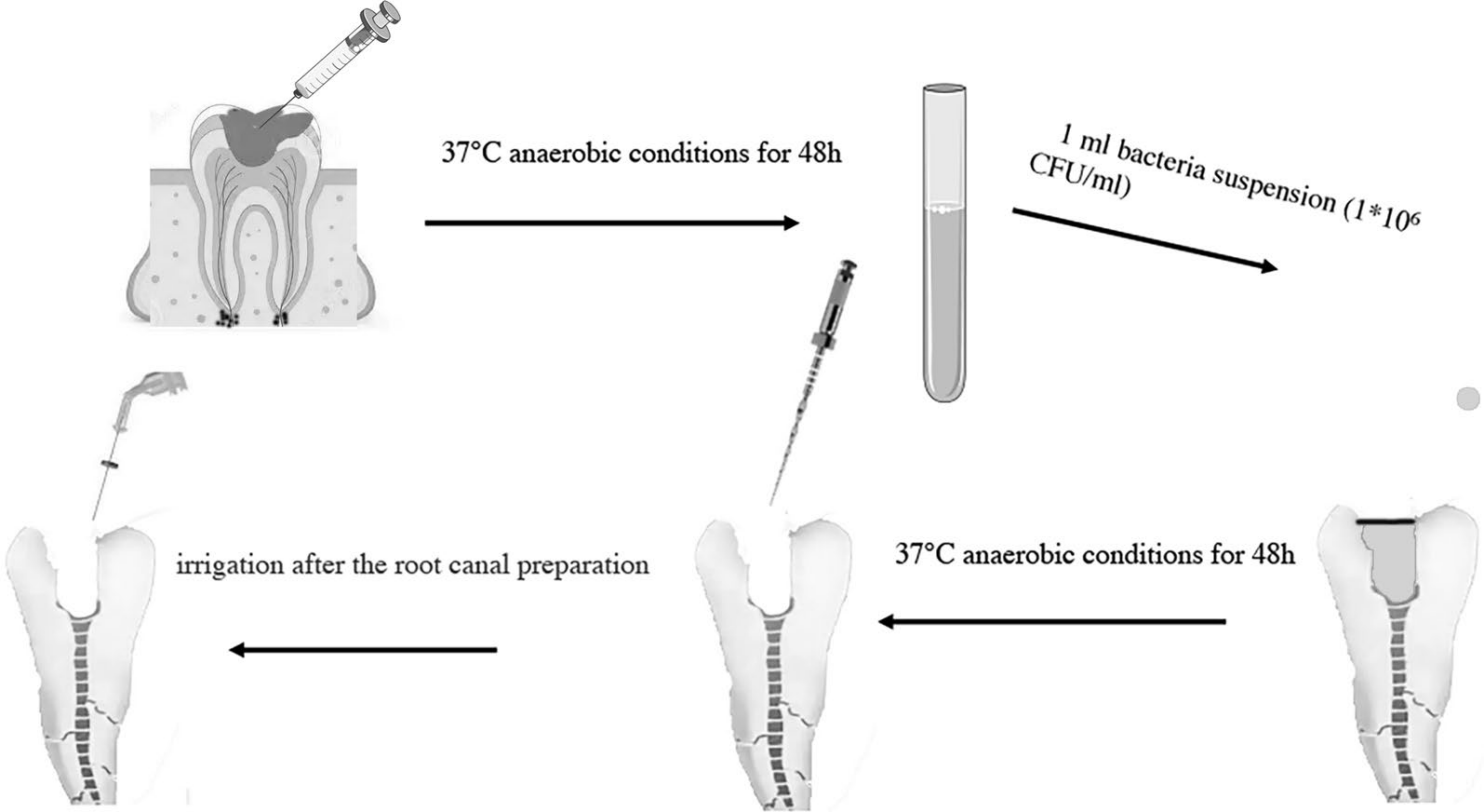
The photographs of the experimental step

Figure 1 presents the photographs of the experimental step. Forty freshly extracted human single-rooted teeth (mandibular premolars) were collected and stored in sterile saline until employed in the experiment. The teeth had straight root canals, confirmed by radiographic examinations, and had been extracted for periodontal reasons. Clinical pathogenic bacteria were grown in brain-heart infusion (BHI) broth at 37 °C with shaking (150 rpm) to form a stationary growth phase suspension of 1 × 10^6^ CFU/mL. 1 mL of this suspension was transferred into each root canal after opening of pulp and the open medullary holes was closed with a fluid resin, and each tooth was placed inside a 1.5-mL microcentrifuge tube that was subsequently sealed, kept upright, and incubated for 72 h at 37 °C under shaking in an anaerobic environment to allow biofilm formation. After 72 h of biofilm formation, the root canals were shaped and prepared using WaveOne Gold Primary (WOG; Dentsply Maillefer) reciprocating files up to the working length by direct vision measurement according to the manufacturer’s instructions, using the X.Smart IQ (Dentsply Sirona) cordless motor. Throughout the instrumentation process, the diameters of the 25-G irrigation needle (side opening) was inserted 1 mm short of the working-length., 100 µg/ml of Pln 149 suspension were used as irrigation solutions in 10 teeth. 5 mL of 3% sodium hypochlorite (n = 10) and 3% hydrogen peroxide (n = 10) were used as positive control group. The physiological saline solution was used as a blank control group (n = 10).

### Detection of biofilms

The biofilms in the infected root canal were first removed by ultrasonic oscillation (JM16D80,Skymen,China) and each sample was washed in 10 mL of phosphate-buffered saline (PBS) solution, and then the bacteria in biofilms were analyzed by quantitative real-time polymerase chain reaction according to our previous methods [15]. The 2 mm diameter root canal specimens were harvested by high speed dental turbines after the irrigation solution for the laser confocal scanning microscopy observation. After fixation, the specimens were stained with LIVE/DEAD® Bacterial Viability Kit (BacLight™) and washed with PBS three times [16]. The biofilms were observed under a laser confocal scanning microscope (Zeiss LSM 710). All the biofilm images were captured and saved using Zeiss ZEN 2010 software. The integrated optical density (IOD) of biofilms was calculated using Image-Pro Plus 6.0 software (Media Cybernetics Inc.)

### MTT assay

Human dental pulp stem cells (hDPSCs, isolated from adult human third molars [17]) were used as test cells since hDPSCs can be easily obtained from teeth extracted for orthodontic reasons. The extraction of DPSCs were conducted according to previous study [18]. MTT (3-(4,5- dimethylthiazol-2-yl)-2, 5-diphenyltetrazolium bromide) colorimetric assay was used to detect in vitro proliferation of hDPSCs according to the previous study [19]. Viable hDPSCs (5 × 10^5^ in 200 µL of RPMI-1640 media) were placed in a 96-well culture plate in the presence and absence of different concentrations of Pln 149 under investigation (100 µg/mL) and incubated at 37 °C in a CO_2_ incubator for 24 h. After removing an aliquot from each well, 10 mM of PBS (180 µL) and MTT (20 µL, 5 mg/mL MTT in PBS) was added, and the plate was incubated at 37 °C for 4 h. Then, an aliquot was removed again, and 200 µL of acidic isopropanol was added to each well. The plate was agitated for 5 min and incubated at 37 °C for 1 h before absorbance values were measured at 570 nm using a titer plate reader [20]. The viability ratio (%) of at each dilution was determined using the following formula:

$$[{\text{Mean}}\;{\text{OD}}\;{\text{of}}\;{\text{treated}}\;{\text{cells}}/{\text{Mean}}\;{\text{OD}}\;{\text{of}}\;{\text{control}}\;{\text{cells}}] \times 100\%$$

### Statistical analysis

All tests were repeated three times with consistent results. SPSS 14.0 software and Image-Pro Plus 6.0 software for Windows was used for data analysis. All data were expressed as means ± SD. The results from the different groups were compared statistically using a Student’s *t*-test or two-way ANOVA. P < 0.05, P < 0.01, and P < 0.001 were considered statistically significant.

## Results

### MBC, MIC, and MTT analysis

**Fig. 2:**
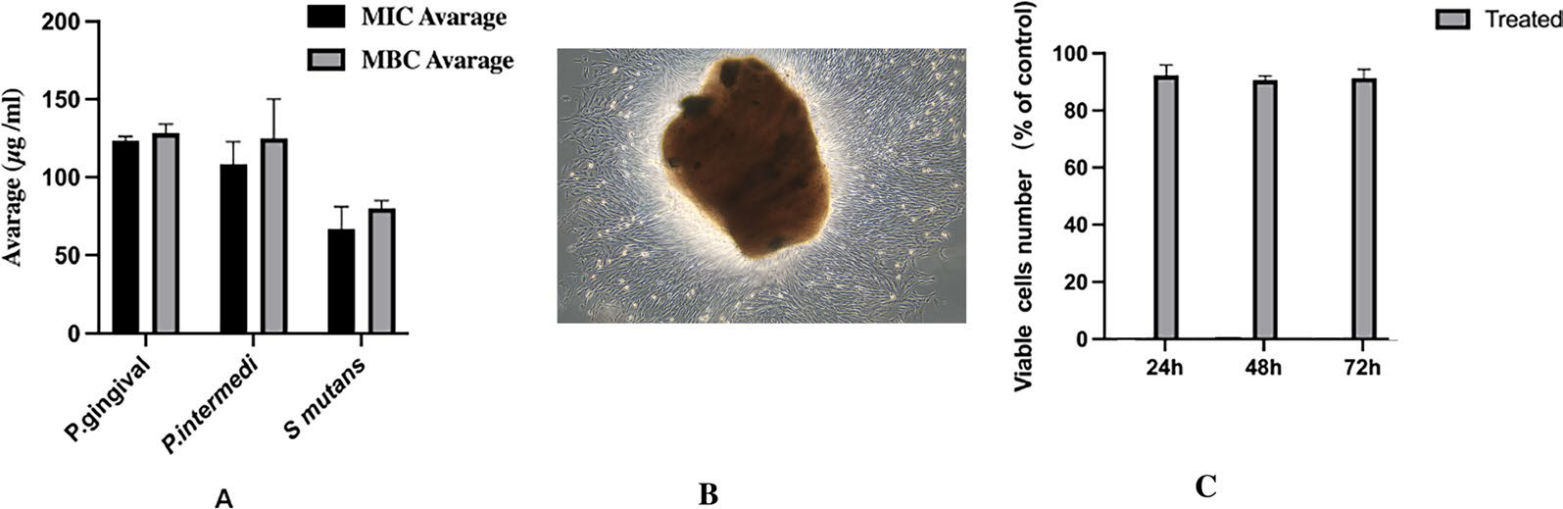
**A** Minimum inhibitory (MIC) and minimum bactericidal concentration (MBC) of Pln 149 with activity against Pg, Sm, and Pi strains; **B** Human dental pulp stem cells (hDPSCs) isolated from dental pulp (P0); **C** MTT assay of hDPSCs

As shown in Fig. 2A, different concentrations of Pln 149 when assessed for Pg, Pi, and Sm showed significant growth inhibition at 60–130 µg/mL, and the MIC of Pg, Pi, and Sm was obtained at 125, 105, and 55 µg/mL. These findings confirm that the MBC of Pln 149 for Pg, Pi, and Sm was effective at 130, 125, and 75 µg/mL. Human dental pulp stem cells (hDPSCs) isolated from dental pulp (P0) was shown in Fig. 2B. After 24, 48, and 72 h of culture, there was no significant difference in hDPSCs viability or proliferation between the Pln 149 and control groups throughout the 24-, 48-, and 72-h culture periods (Fig. 2C).

### Inhibition rates and diversity changes of pathogenic bacteria in root canals

**Fig. 3:**
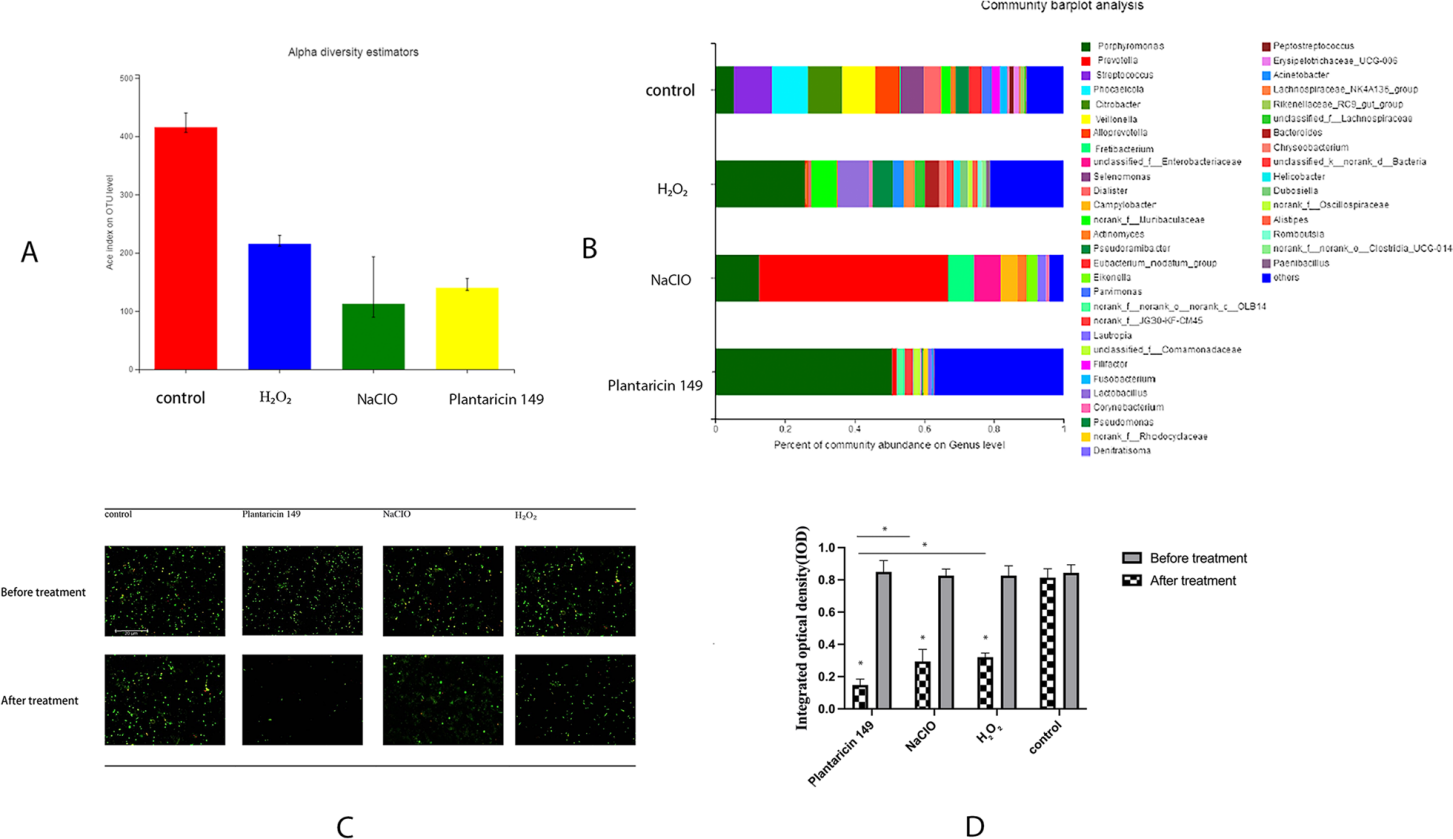
**A** Alpha diversity of estimators of sodium hypochlorite, hydrogen peroxide, Pln 149 suspension and control groups; **B** Pecent of community abudance in gene levels in sodium hypochlorite, hydrogen peroxide, Pln 149 suspension and control groups; **C** Confocal laser image of biofilms in sodium hypochlorite, hydrogen peroxide, Pln 149 suspension and control groups before and after treatment; **D** IOD of biofilms in sodium hypochlorite, hydrogen peroxide, Pln 149 suspension and control groups before and after treatment (*P < 0.05)

qPCR Primers used in this study were shown in Table 1. Table 2 presents the inhibition rates in the root canals before and after treatment. Pln 149 suspensions showed strong inhibition rate of Pg, Pi, and Sm in root canals after root canal irrigation compared with NaCIO, H_2_O_2_ (P < 0.05). Figure 3A and B show the diversity and community changes in the four study groups. As shown in Fig. 3 A, Pln 149 reduced the diversity in root canals compared with control groups significantly. Pln 149 reduced the species of community in infected root canals significantly compared with NaCIO, H_2_O_2_ and control groups and Pg, Pi, and Sm were still the main pathogens in infected root canals (Fig. 3B).

**Table 1:** qPCR Primers used in this study

Species	Sequence (5’ to 3’)	Target gene	Amplicon size (bp)
*S. mutans*	F:AGTGCCAAGACTGACGCTTT	*dexA*	141
	R:GGGCTGACTGCTTCTGGAGT		
*P. intermedia*	F: TTFGTrGGGGAGTAAAGCGGG	*tetQ*	575
	R:一TCAACATCTCTGTATCCTGCG		
*P. gingival*	F:GGAAGAGAAGACCGTAGCACAAGGA R: GAGTAGGCGAAACGTCCATCAGGTC	*rpoB*	143
*Total Bacteria*	F:CCATGAAGTCGGAATCGCTAG	*16 S rRNA*	89
	R:GCTTGACGGGCGGTGT

**Table 2:** The detectable quantity and variety of pathogenic bacteria in root canal before and after treatment (means ± SD)

		*P.gingival*	*P.intermedia*		*S.mutans*
Plantaricin 149	Before treatment	6.25 ± 0.43	4.34 ± 0.67		4.54 ± 1.21
	After treatment	3.09 ± 0.58	1.97 ± 0.43		2.31 ± 0.56
	Inhibiting rates	52.16 ± 9.28%^ab^	54.60 ± 8.98%^ab^		49.11 ± 9.05%^ab^
NaCIO	Before treatment	7.08 ± 1.97	5.09 ± 1.13		4.43 ± 0.98
	After treatment	5.48 ± 1.54	3.78 ± 1.34		2.98 ± 0.87
	Inhibiting rates	22.59 ± 6.74%	25.73 ± 4.78%		32.73 ± 6.89%
H_2_O_2_	Before treatment	6.98 ± 2.01	4.89 ± 1.76		4.21 ± 1.12
	After treatment	5.97 ± 1.87	4.01 ± 0.89		3.78 ± 0.98
	Inhibiting rates	14.46 ± 3.32%	17.99 ± 3.88%		10.21 ± 2.98% ^ab^

Values are given in 10 ^6^CFU/ml

^a^means P < 0.05 compared with NaCIO, ^b^means P < 0.05 compared with H_2_O_2_

### Biofilm formation in infected root canals

Figure 3 C and D show the biofilm formation under the laser confocal scanning microscope (CSLM ) stained with LIVE/DEAD® Bacterial Viability Kit. CSLM clearly indicated that all groups exerted the same bacteria-rich biofilms before treatment. After treatment, Pln 149 suspension, NaCIO and H_2_O_2_ groups all inhibited the biofilm significantly compared with control groups(P < 0.05). Meanwhile, Pln 149 suspension reduced the biofilm formation significantly after root canal irrigation compared with NaCIO and H_2_O_2_ groups (P < 0.05) under CLSM. IOD results of the four groups followed the same trend with confocal scanning microscope images.

## Discussion

The bacterial debris in the root canal can be flushed through the root canal. Appropriate irrigation solutions’ physical and chemical flushing action with excellent bacterial inhibition and sterilization effect can effectively remove the debris from the fine root canal(s) of the affected tooth to significantly improve treatment outcomes [21]. Although the presence of the smear layer can hinder the external bacteria to a certain extent, the more critical function is to protect the internal bacteria from being removed [22]. Therefore, when irrigating the root canal, we should try our best to use anti-biofilm root canal irrigation solution for efficiently removing the internal and external bacterial biofilm.

When NaClO solution is used to irrigate the affected root canal, a reduction reaction can also take place to form an HClO oxidant network [23]. HClO is a strong oxidant that can remove anaerobic bacteria and microorganisms from the root canal. The concentration of NaClO solution is positively correlated with the concentration of HCl solution. HClO solution can dissolve organic matter and necrotic tissues in the root canal. However, there is a problem with the HClO solution. If its concentration is too high, it will strongly irritate the patient’s oral mucosa and destroy the micro-strength of teeth [24]. Hydrogen peroxide is still one of the most widely used root canal irrigation solutions in primary stomatology [25]. It has the effects of oxidation, deodorization, sterilization, and oxidative foaming. It is of great significance for removing necrotic tissues from the root canal; however, hydrogen peroxide is less effective in preventing the formation of bacterial biofilms in the root canals. *Lactobacillus plantarum* is one of the most common *lactobacilli* that could produce an antibacterial protein called bacteriocin [26]. As a natural substance with antibacterial activity, bacteriocin has gradually attracted attention and has become a research hotspot because of its potential to replace antibiotics. Bacterial infection is an important pathogenic factor for chronic periapical periodontitis. Reinfection of the root canal after treatment is one of the main reasons for the failure of root canal treatment [27]. *Porphyromonas pulposus*, *Porphyromonas gingivalis*, and *Prevotella intermedia* are Gram-negative, obligate anaerobic bacteria and are the dominant pathogens in root canal infections [28]. Most of them are isolated from infected root canals and periapical abscesses. They are almost found only in infected root canals, with a high detection rate, and are considered to be the core bacteria in the infected root canal. Our previous research showed that Pln 149 exhibited strong antibacterial activity against *P. gingivalis*, indicating its potential value in root canal irrigation [29].

Root canal biofilm is the principal cause of endodontic treatment failure [30]. Severe endodontic infection caused by pulp necrosis, long-term exposure of the pulp cavity, and failure of root canal treatment are the premise of root canal biofilm formation [31]. Pulpal inflammatory lesions extend continuously or suddenly to the root tip. Inflammatory exudate provides a liquid environment for biofilm formation, conducive to the invasion and reproduction of planktonic bacteria in the root canal, attachment to the root canal wall, and formation of root canal biofilm around the bacterial inflammatory interface [32]. Host proteins and viscous substances produced by bacteria provide a material basis for forming and maintaining bacterial aggregates. Therefore, elimination of the root canal biofilm is the key to the success of root canal treatment. Recent research has shown that the bacterial population in the biofilm works like a ‘bacterial brain,’ which means bacteria could communicate with each other through ion channel-mediated electric signals [33]. Our previous study showed that Pln 149 could inhibit ion channels [29], and in our current experiment, Pln 149 showed strong anti-biofilm effects on root canal biofilms. With no cytotoxicity to human dental pulp stem cells, Pln 149 offers promising applications as an irrigation solution and disinfection medicament in root canals.

In our present study, we only tested the pathogenic bacteria from only one person; however, the etiology of infection of root canal may be more complicated for the bacteria in each infection are different, meanwhile it is difficult to simulate root canal infection in vitro. Therefore, the results require further research. In the future, we will also consider all aspects to design experiments in vivo.

In conclusion, considering the excellent antimicrobial effects and low cytotoxicity, Pln 149 might be a promising option for root canal irrigation solutions, and with a good application value in root canal treatment.

## Data Availability

The authors confirm that the data supporting the findings of this study are available within the article and the datasets used during the current study are available from the corresponding author on reasonable request.
